# Osteomyelitis Infection of *Mycobacterium marinum*: A Case Report and Literature Review

**DOI:** 10.1155/2015/905920

**Published:** 2015-01-18

**Authors:** Hao H. Nguyen, Nada Fadul, Muhammad S. Ashraf, Dawd S. Siraj

**Affiliations:** Division of Infectious Diseases, Department of Internal Medicine, The Brody School of Medicine at East Carolina University, Mailstop 715, Doctors Park 6A, Greenville, NC 27834, USA

## Abstract

*Mycobacterium marinum* (*M. marinum*) is a ubiquitous waterborne organism that grows optimally at temperatures around 30°C. It is a nontuberculous *Mycobacterium* found in nonchlorinated water with worldwide prevalence. It is the most common atypical *Mycobacterium* that causes opportunistic infection in humans. *M. marinum* can cause superficial infections and localized invasive infections in humans, with the hands being the sites most frequently affected. It can cause skin lesions, which are either single, papulonodular lesions, confined to an extremity, or may resemble cutaneous sporotrichosis. This infection can also cause deeper infections including tenosynovitis, bursitis, arthritis, and osteomyelitis. Disseminated infections and visceral involvements have been reported in immunocompromised patients. We here report a case of severe deep soft tissue infection with necrotizing fasciitis and osteomyelitis of the left upper extremity (LUE) caused by *M. marinum* in an immunocompromised patient.

## 1. Introduction

*Mycobacterium marinum* was first isolated in 1926 by Aronson from salt water fish carcasses in the Philadelphia aquarium [1]. Baker and Hagan discovered that the mycobacterium caused tuberculosis in fresh water platyfish and called it* M. platypoecilus* [2]. In 1951, it was recognized as a human pathogen by Linell and Norden who isolated it from the skin lesions of swimmers from the swimming pool in Sweden [3].

*M. marinum* is a nontuberculous mycobacterium belonging to Runyon group I, a photochromogen [4].* M. marinum* has a worldwide distribution and primarily infects fish that can secondarily contaminate aquaria, swimming pools, rivers, and seawater. When transmitted to animals such as amphibians, fish, mice, and bats, it can be highly prevalent in fish tanks and cause infections and death in various fish species [4]. The colonies form a yellow pigment when the culture medium on which they are growing is exposed to light. Optimal growth is at 30 to 32°C. It grows slowly or not at all at 37°C. This organism will grow nicely on standard mycobacterial media and produces smooth and shiny colonies in an average of 10 to 28 days, though the cultures should be held for 6 weeks if negative.* M. marinum* preferentially grows at cold temp between 30 to 32°C and thus, infections with* M. marinum* are usually localized primarily to the skin. Less commonly it extends to involve deeper structures such as joints and tendons. Dissemination has been reported, but is distinctly unusual [5]. In general,* M. marinum *infection in humans is comparatively rare. The approximate annual incidence in the United States is 0.27 confirmed cases per 100,000 inhabitants [6]. In 90% of cases, infection takes place via trauma to the upper extremity and is not transmittable from person to person [7].

We searched the published English literature for cases of severe deep soft tissue infection and osteomyelitis caused by* M. marinum* both in immunocompromised and immunocompetent individuals. We found eleven published cases of adult* M. marinum* complicated with osteomyelitis. Four of them had compromised immune systems. This case review aims to focus on severe deep soft tissue infection and osteomyelitis caused by* M. marinum*.

## 2. Case Report

**Figure 1:**
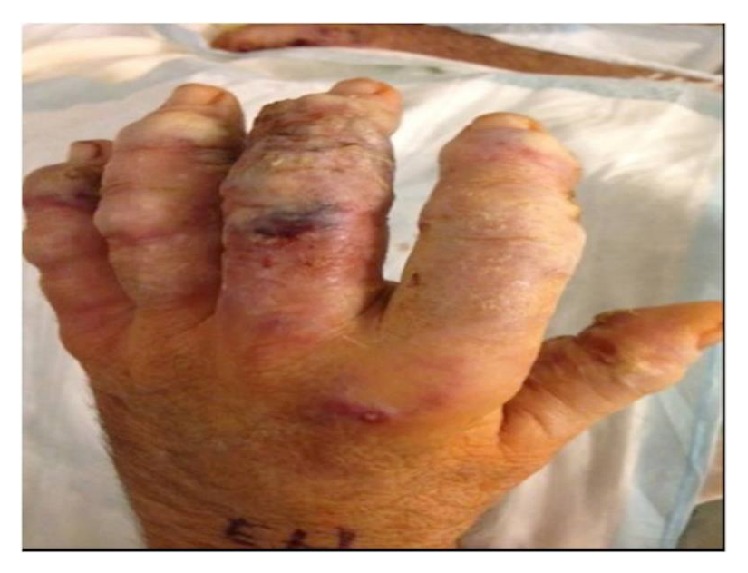
Inflammatory nodules on the left hand on second admission.

Patient is a 64-year-old Caucasian male presented to the emergency room at the end of December at our facility with worsening left arm swelling. Two weeks ago, he was initially admitted for the right arm swelling and tenderness. He, at baseline, has been on leflunomide for three years for treatment of rheumatoid arthritis and previously was on prednisone for the same reason. Patient reported that, in mid-November, he started to experience significant pain in both wrists and was evaluated at the rheumatologist office for possible rheumatoid arthritis flare. He had received steroid injections in bilateral wrists. During his first admission two weeks prior, he was diagnosed with deep abscesses of right upper extremity (RUE) which has later gotten incision and drainage. At that time, only bacterial cultures were sent, which grew methicillin-sensitive* Staphylococcus aureus*. Patient was discharged home on trimethoprim/sulfamethoxazole oral tablets but readmitted seven days later with pain and pustules now mainly on all of the fingers of the left forearm (Figure 1).

**Figure 2:**
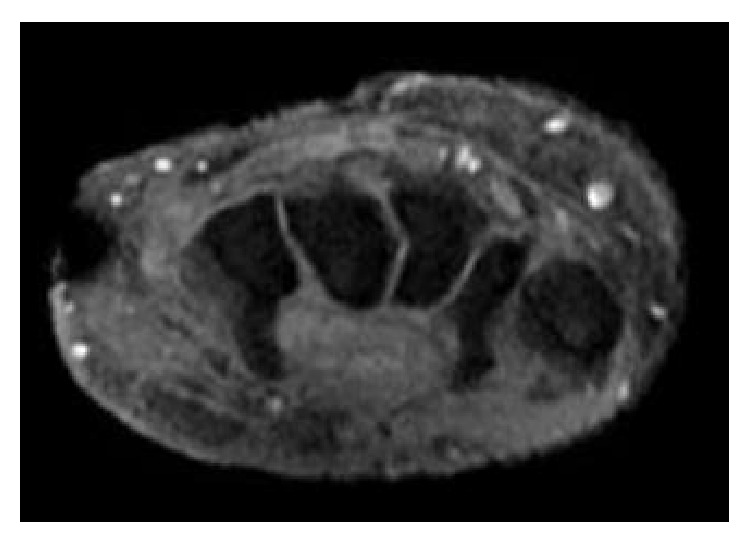
MRI of the left arm showed multiple ulcerations extending from skin into subcutaneous fat with appearance concern for necrotizing fasciitis and osteomyelitis.

**Figure 3:**
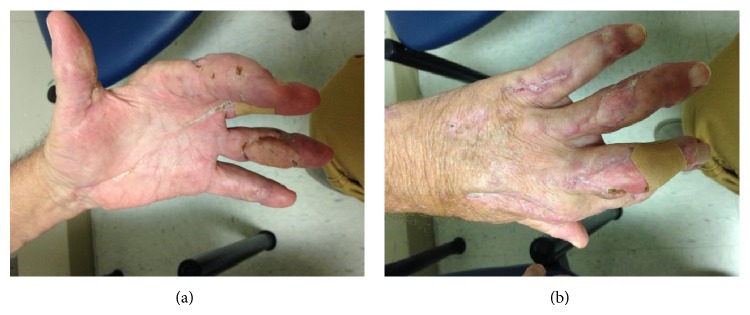
Patient's left hand at his 9-month follow-up.

He again underwent drainage and bacterial and acid fast bacilli smear and cultures were sent.* M. marinum* was identified phenotypically in our lab when the culture grew at 30 degrees Celsius. The sample was also sent out for biochemical sequence and further identified as* M. marinum* (Nichols Institute, Chantilly, VA). He was empirically treated with intravenous imipenem, linezolid, and azithromycin. During this second admission, magnetic resonance imaging (MRI) of the left arm was done which showed multiple ulcerations extending from the skin surface into the subcutaneous fat with the largest visualized at the palmar aspect of the wrist, extending deep to the level of the carpal tunnel/hook of the hamate, at the dorsal and ulnar aspects of the mid- and distal forearm at two sites and at the ulnar aspect of the distal forearm suggesting tenosynovitis with necrotizing fasciitis. He recovered well from the surgery and was discharged on doxycycline and azithromycin while waiting for susceptibility. Despite being adherent to this regimen for four weeks, he was ultimately readmitted a third time with new abscess formation on medial aspect of his proximal LUE and repeated MRI showing osteomyelitis of the left distal ulna (Figure 2). Susceptibility test with minimal inhibitor concentration (MIC) (Focus Diagnostics, Inc., Cypress, CA) came back with* M. marinum* resistant to doxycycline and rifampin (Table 1). He underwent the third incision and drainage with the skin graft for his LUE. Therapy was then changed to azithromycin and trimethoprim/sulfamethoxazole based on the susceptibility result (Table 1). Because of extensive disease and slow improvement on therapy, our patient received nine months of antibiotics therapy with good response (Figure 3). He is still being followed by infectious diseases and plastic surgery.

## 3. Methods

We searched the English language literature published until August 2014 in the PubMed database. Relevant studies were identified using various key word combinations including “mycobacterium,” “marinum,” “osteomyelitis,” and “treatment.” No lower publication date limit was set. Eleven published cases of* Mycobacterium marinum* osteomyelitis were ascertained. The clinical characteristics including age, gender, predisposing factors, duration of therapy, clinical outcome, and list of antimicrobials used were summarized in Table 2.

## 4. Discussion

*Mycobacterium marinum* is a nontuberculous mycobacterium belonging to Runyon group I, a photochromogen. From the literature review, its infection occurs approximately about 2–6 weeks after direct inoculation of the organism either from fish fins and bites or from the handling of aquariums. Incubation period is normally about 2–6 weeks. However, there are some cases reporting an incubation time of 2 to 4 months and longer, with some cases reporting an incubation period as long as 9 months due to the slow-growing nature of this organism [6].

There are many different clinical presentations of* M. marinum* infection. In immunocompetent patient, most commonly it appears as a solitary papulonodular lesion on an extremity. In particular, these lesions tend to occur over a prominence that has a predisposition to be abraded, such as finger, hand, or knee. A history of preceding minor trauma is common and an occupation or hobby that resulted in a likely environmental water exposure is the rule [18]. Inflammatory nodules or abscesses can develop in severely immunosuppressed patient, usually in a sporotrichotic type of distribution [3]. This “sporotrichoid” disease begins with distal inoculation and may lead to the development of nodular lymphangitis. Over a period of months, localized cutaneous disease can spread to deeper soft tissues, causing tenosynovitis, arthritis, bursitis, and/or osteomyelitis of the underlying bone; it can also be life-threatening, and lesions may or may not be painful [6]. Infections with* M. marinum* can be theoretically classified into four different clinical categories to help in guiding treatment options. Type I includes single or limited (1–3 lesions) superficial cutaneous infections (ulcerated, crusted, or verrucous plaques or nodules). Type II includes numerous (>3) lesions in a sporotrichoid distribution pattern or with inflammatory nodules, abscesses, and granulomas. Type III includes deep infections with or without skin involvement, including tenosynovitis, arthritis, bursitis, and/or osteomyelitis and Type IV refers to disseminated infection, lung involvement, and other systemic manifestations. Bacteremia is usually seen in immunocompromised patients but is considered very rare [19, 20]. Our case was the clinical manifestation of Type III infection.

In the literature, there are eleven cases where* M. marinum* presented with deep tissue infection and osteomyelitis. The first case was published in 1972 involving a 36-year-old healthy Vietnamese female who was exposed to salt water and fishing presented with deep infection and osteomyelitis of the hand leading to amputation. Among the eleven cases, there were seven cases of immunocompetent patients and four cases with underlined immunocompromised status as listed in Table 1. The majority of infections involved the upper extremities. Ten out of eleven cases were treated with both chemotherapy and extensive debridement, and four of them required amputation. None of the eleven cases had Type IV or disseminated infection nor bacteremia.

Diagnosis of* M. marinum* infection is usually delayed, suggesting that most physicians are not familiar with the disease [8]. This is probably because of the rareness of the infection and a failure to establish a history of exposure to aquatic environments or to tropical fish. Key diagnostic elements for* M. marinum* infections are a high index of suspicion raised by negative bacterial tissue cultures, poor response to conventional antibiotic treatments, and a history of aquatic exposure [6, 18]. A definite diagnosis is confirmed by isolation and identification of the organism. All of the cases reported listed in Table 1 had positive cultures for* M. marinum* and the majority of the cases had history of exposure to aquatic environments.

However, in practice, the diagnosis remains largely presumptive, based on clinicohistological features and the response to appropriate antimicrobial treatment, regardless of culture results [6]. In addition, polymerase chain reaction (PCR) allows the early detection of the organism from a biopsy specimen. This technique may prove to be helpful and supersede conventional methods in the rapid diagnosis and species identification of nontuberculous infections and become the test of choice in the future [9].

Occasional spontaneous resolution of soft tissue infection by* M. marinum* has been reported in the literature. The main purpose of therapy aims for a rapid recovery from the infection and the prevention of progression to deeper structures [5]. Monotherapy is usually applied for skin and soft tissue infection, but this is considered ineffective for deeper structure infections. Among eleven cases with deep seated infection, only one was treated with Doxycycline monotherapy for six months (Table 2).

There have been no comparative trials of different treatment regimens for soft tissue infection by* M. marinum*. A literature review that was published in 2007 suggests that topical therapy as a sole treatment is completely ineffective and unnecessary [6]. In limited superficial cutaneous infections (Type I), the second-generation tetracycline minocycline (100 mg b.i.d.), clarithromycin (500 mg b.i.d.), doxycycline (100 mg b.i.d.), and trimethoprim/sulfamethoxazole (800 mg b.i.d.), each as monotherapy, are considered effective treatment options.

Based on the available literature review, a reasonable approach is to treat patient with two active antimicrobials for at least one to two months after resolution of symptoms [5–7]. This is more so in immunocompromised individuals and cases of severe cutaneous infections (Type II or III). Typical duration of therapy is 3-4 months. Duration of treatment is usually longer in patients with deeper structure infections [6]. Because of the extensive involvement and slow recovery, our patient treatment has been extended for nine months. In our literature review, the longest duration of therapy documented was for 18 months (Table 2).

*M. marinum* is usually susceptible to rifampin, rifabutin, ethambutol, clarithromycin, sulfonamides or trimethoprim/sulfamethoxazole, doxycycline, and minocycline [4, 5, 7]. In the literature, the most commonly used combinations include clarithromycin and rifampin, clarithromycin and ethambutol, or the combination of ethambutol and rifampin. In our patient,* M. marinum* was resistant to rifampin and unfortunately could not be used as one of the agents in the combination regimen (Table 1). However, our patient showed good response with the therapy of azithromycin and trimethoprim/sulfamethoxazole combination.

In cases with severe cutaneous infections (Types I–III), surgical treatment may be required if the infection has not been controlled by chemotherapy. Deeper infections (Type III) may require prolonged systemic treatment and repeated surgical debridement. However, the selection of cases and the time of surgical intervention require good judgment. In disseminated infection or bacteremia (Type IV), combined (antimicrobial plus antimycobacterial) intravenous therapy of three drugs may be required [6, 9, 10].

The use of isoniazid, streptomycin, and pyrazinamide as empirical treatment options should be avoided, as the resistance of the organism to these agents is well documented [6, 21]. Cryotherapy, X-ray therapy, electrodesiccation, photodynamic therapy, and local hyperthermic therapy have also been proposed as therapeutic alternatives, but there is so far no solid evidence on the successful cure rate on these [21].

In conclusion, infections due to* M. marinum *are uncommon, but not rare.* M. marinum* infection should always be included in the differential diagnosis of all cases with poorly healing wounds in upper extremities and in persons with a history of exposure to aquariums. The diagnosis requires both a detailed history and sophisticated microbiological and PCR-based investigations. No large systemic studies have been performed to determine the optimal treatment regimen. In most cases a combination of antibacterial drugs should be given as well as long-term therapy depending on the duration and severity of infection.

## Figures and Tables

**Table 1:** Susceptibility testing result of *M. marinum* in our patient.

Organism	*M. marinum * with minimal inhibitory concentration (MIC) values
Amikacin	8 susceptible (S)
Ciprofloxacin	8 resistance (R)
Clarithromycin	2 S
Doxycycline	16 R
Ethambutol	4 S
Ethionamide	2.5
Isoniazid	>8
Linezolid	4
Moxifloxacin	4 R
Rifampin	2 R
Rifabutin	≤0.25 S
Streptomycin	32
Trimethoprim/sulfamethoxazole	2/38 S

**Table 2:** Cases of *M. marinum* osteomyelitis in the literature review with treatment and clinical outcome.

Author	Year	*n*	Age	Sex	Immune status	OM site	Source	Chemotherapy + surgical debridement	Duration	Outcome
Jolly and Seabury [8]	1972	1	36	M	Normal	Finger	Fishing	None	N/a	Amputation
Wendt et al. [9]	1986	1	47	F	Normal	Finger	Unknown	INH, rifampin, and ethambutol	3 weeks	Amputation
Clark et al. [10]	1990	1	56	M	Normal	Finger	Fishing/steroid injection	Minocycline, rifampin, and ethambutol	9 months	Recovered
Vazquez and Sobel [11]	1992	1	62	F	Normal	Finger	Fish tank	INH, rifampin, and bactrim	3 weeks	Amputation
Harth et al. [12]	1994	1	56	M	Normal	Finger	Fish tank/steroid injection	Ciprofloxacin, ethambutol, and rifampin	12 months	Recovered
Alloway et al. [13]	1995	1	71	M	Normal	Finger	Fishing	Ciprofloxacin, ethambutol, and rifampin	12 months	Recovered
Barton et al. [14]	1997	1	48	F	Deficient	Finger	Fish tank	Doxycycline	6 months	Recovered
Shih et al. [15]	1997	1	52	F	Normal	Finger	Fish dealer	Clarithromycin and ethambutol	18 months	Recovered
Wilson et al. [16]	2003	1	47	M	Deficient	Foot (talus)	None	Rifabutin and ciprofloxacin	3 months	Amputation
Sivan et al. [17]	2008	1	66	M	Deficient	Leg	Fish tank	Rifampicin, ethambutol, and moxifloxacin	12 months	Recovered
Present case	2014	1	64	M	Deficient	Arm	Fishing	Azithromycin and bactrim	9 months	Recovered

Barton et al., 1997 [14]: immunosuppressive therapy for rheumatoid arthritis and fibrosing alveolitis.

Wilson et al., 2003 [16]: acquired immunodeficiency syndrome.

Sivan et al., 2008 [17]: immunosuppressive therapy for bullous pemphigoid.

Present-2014: immunosuppressive therapy for rheumatoid arthritis.
